# Symmetry breaking organizes the brain’s resting state manifold

**DOI:** 10.1038/s41598-024-83542-w

**Published:** 2024-12-30

**Authors:** Jan Fousek, Giovanni Rabuffo, Kashyap Gudibanda, Hiba Sheheitli, Spase Petkoski, Viktor Jirsa

**Affiliations:** 1 INSERM, INS, Institut de Neurosciences des Systèmes, Aix Marseille University, 13005 Marseille, France; 2 Central European Institute of Technology (CEITEC), Masaryk University, Brno, Czech Republic; 3 Department of Neurology, University of Minnesota, Minneapolis, MN USA; 4 Department of Psychiatry and Behavioral Sciences, University of Minnesota, Minneapolis, MN USA

**Keywords:** Dynamical systems, Network models

## Abstract

Spontaneously fluctuating brain activity patterns that emerge at rest have been linked to the brain’s health and cognition. Despite detailed descriptions of the spatio-temporal brain patterns, our understanding of their generative mechanism is still incomplete. Using a combination of computational modeling and dynamical systems analysis we provide a mechanistic description of the formation of a resting state manifold via the network connectivity. We demonstrate that the symmetry breaking by the connectivity creates a characteristic flow on the manifold, which produces the major data features across scales and imaging modalities. These include spontaneous high-amplitude co-activations, neuronal cascades, spectral cortical gradients, multistability, and characteristic functional connectivity dynamics. When aggregated across cortical hierarchies, these match the profiles from empirical data. The understanding of the brain’s resting state manifold is fundamental for the construction of task-specific flows and manifolds used in theories of brain function. In addition, it shifts the focus from the single recordings towards the brain’s capacity to generate certain dynamics characteristic of health and pathology.

## Introduction

The human brain at rest exhibits a remarkable richness of neural activity structured both in time and space. Early computational modeling studies explored how these spontaneous fluctuations are constrained and how their organization is shaped by the anatomic connectivity^1–4^ enabling to start disentangling the mechanisms of the resting state dynamics in silico. A substantial body of work has related the emergent activity patterns at rest to the brain functional networks involved in task conditions^5,6^, and shown that the spatiotemporal variability of resting-state activity possesses functional significance^7–9^, relevance to cognitive task performance^10^, consciousness levels^11^, changes during aging^12,13^, mental disorders^14^, and neurodegenerative diseases (e.g. Alzheimer’s dementia;^15^). The structure of the resting state dynamics changes over time^16^ and is characterized by a range of properties such as metastability^17,18^, event-like coactivations^19–21^ and traveling waves^22^. However, our understanding of the mechanisms underlying these spatiotemporal patterns of the brain activity at rest is still incomplete^23^ and whole brain network models have a crucial role to play on that front^24^.

There is general agreement that the resting brain operates near criticality^25^. This is supported by a large range of analyses performed on simulated and empirical data using network-based measures (functional connectivity, functional connectivity dynamics), information theoretical measures (entropy, ignition), and descriptions of spatiotemporal dynamics (avalanches, cascades). Modeling efforts provide further evidence for the close relationship between the empirical data features and the properties of the structural network, local dynamics, coupling strength, neural gain^4,13,26–31^. The resting state dynamics can then be understood as noise-driven fluctuations of brain activity, operating near criticality and constrained by the brain connectivity^2,32^. However, the models above leave a gap in the description of a mechanism, as the relations between the causal properties of the model and the dynamical signatures are left implicit. The mechanistic description in terms of the generative system can be thus further improved by showing how the properties and components of the model give rise to the features of the generated activity. It requires formulation in terms of causal activities of their constituent entities and renders the end stage, in our context the resting state dynamics, intelligible by showing how it is produced^33^. To explain is thus not merely to redescribe one regularity (e.g. functional connectivity dynamics, or maximization of entropy) as a series of several (such as near-criticality, cascades, ignition). Rather, explanation involves revealing the productive relation between causal activities linked to their constituent entities.

**Fig. 1:**
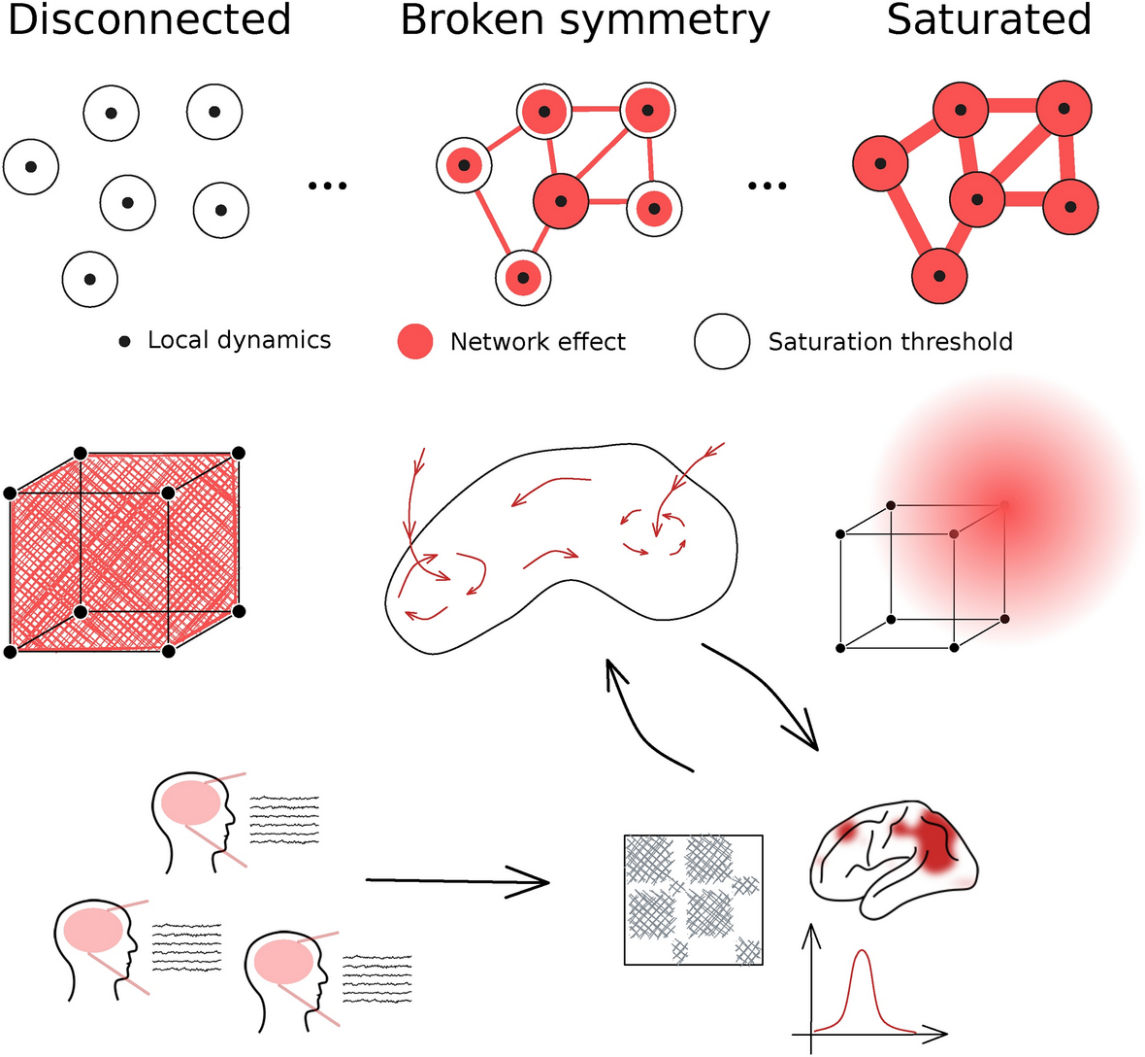
Structured flows on manifolds as the focus of resting state characterization. For the structure of the connectivity of the dynamical system, we consider the spectrum defined by the two symmetrical limit cases: a fully disconnected network, and a network where the external input dominates the activity at each node. Driven by noise, the disconnected system exhibits fully statistical, high-dimensional dynamics - it explores the whole state space in an equidirectional manner. On the other hand, the dynamics of the strongly connected system are equally high-dimensional as the system is constrained by the high external input to a subspace which it explores again equidirectionally driven by the sum of the noisy network inputs. The dynamics of the sparsely connected system lead to an object in between—a low-dimensional attractive manifold with an associated flow (SFM). It is this object we wish to put in the center of interest and characterize. While the SFM object remains the same, connections are made to data of various modalities with the help of suitable data features.

In this paper we aim to remedy this situation and provide this explanation using Structured Flows on Manifolds (SFMs)^34–38^. SFMs is a mathematical framework explaining how low dimensional dynamics, reflecting generative sets of rules underlying behavior, emerges in high-dimensional nonlinear systems, specifically dynamical systems on networks modeling macroscale brain dynamics. The structural connectivity plays an important role here by providing symmetry constraints to the range of viable solutions of the system’s equations, which in turn collectively define the dynamic repertoire of the system^35^. When properly linked to the network’s constituent entities (functional nodes and connectivity), we will demonstrate how their causal activities lead to the formation of the brain’s resting SFM, comprising all its dynamic signatures (see Fig. 1), thus closing the gap between the mechanisms of the generative model and the observed functional features. If we distill the previous reports of brain resting state data analysis from the dynamical systems point of view, we arrive at the following main empirical signatures that should be part of the end stage of a successful mechanistic description: bistability of single region activation^39–41^, low-dimensionality of the global system dynamics in state space^7,42,43^, cascade propagation^44^, multistability of recurrent coactivation spatial patterns^18,45^ and their non-trivial temporal dynamics or intermittency^21,32,46^. These signatures will constitute the key features of what we will describe as structured flows on the low-dimensional resting state manifold. We assess the manifold and the associated flow using local and global unfolding using principal component analysis (PCA), thus side-stepping the need for a closed-form solution. Such a solution would be not only difficult to derive, but also limited in terms of generalization to other models and application to empirical data.

## Results

In what follows we employ whole-brain modeling to study the low dimensional manifold and the associated structured flows of the spontaneous resting state dynamics, and how these relate to the structural connectome. We constructed a brain network model (BNM) in The Virtual Brain^47^ using the two-dimensional mean-field model of an ensemble of quadratic integrate-and-fire neurons (^48^; MPR) to govern the regional dynamics coupled with a connectome derived from a subject from the Human Connectome Project^49^. We applied the Balloon-Windkessel model^50^ to the simulated neuronal mass activity to generate realistic BOLD signals. From these, we calculated the dynamic Functional Connectivity (dFC), which captures the time-dependent variations of the Functional Connectivity (FC), and is computed either as correlation between FCs extracted in a sliding window (windowed $dFC_w$), or as the correlation between the time-points of the edge time series (edge $dFC_e$). We evaluate the dFC in terms of *fluidity* of the system’s dynamics—that is the propensity to dwell in specific brain states (defined by the FC) and shift and return between several such states (see Section "Functional connectivity dynamics" for more details). The fast neuronal activity is decomposed in a 2N-dimensional state space using Principal Component Analysis (PCA) to unveil the low-dimensional manifold on which the system evolves.

When driven by noise, the network of the bistable MPR nodes has the capacity to exhibit realistic dFC supported by neuronal cascades when the network input is scaled appropriately^44^ — we refer to this regime as the *working point*. The noise together with the network input drives the switching between the up- and down-state of the individual nodes, while the network mediates the coordination reflected in the functional connectivity. In the following sections, we explore how the manifold of the resting state activity arises from the networked interactions, how it shapes the multistability of the functional connectivity in the simulated BOLD, and how it relates to empirical observations.

### Symmetry breaking: working point for dFC

Symmetry breaking by the network connectivity may give rise to the low-dimensional behavior of an otherwise high-dimensional nonlinear system^35^. We start the analysis here by verifying this assumption, and evaluate whether this low-dimensional regime also coincides with increased fluidity of the spontaneous dynamics.

**Fig. 2:**
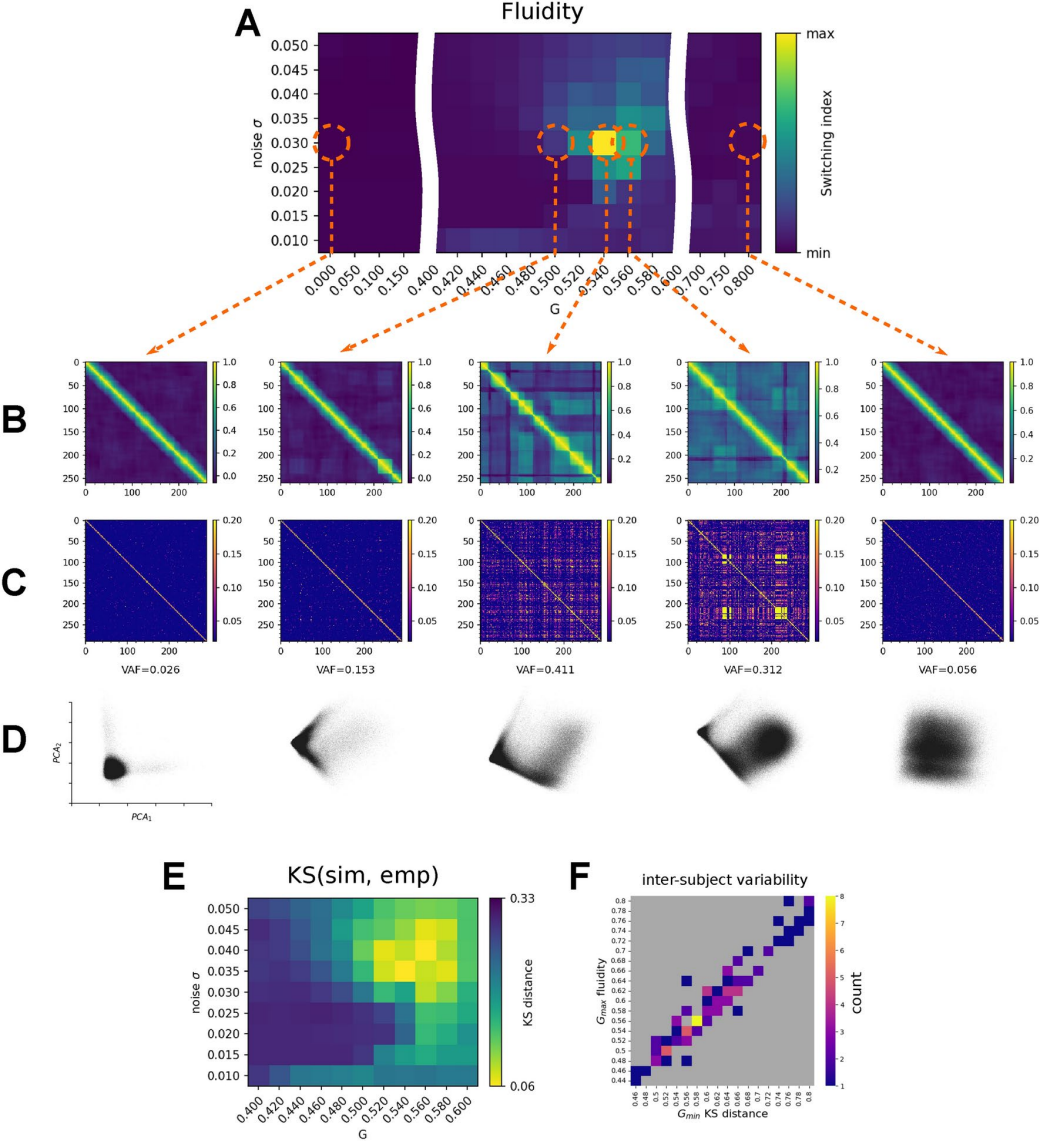
Brain network model and symmetry breaking. The brain network model is simulated for varying levels of global coupling parameter *G* and noise variance $\sigma$ to produce both time series of the state space variables $\textbf{r}(t), \textbf{V}(t)$, and the BOLD signal. For each combination of *G* and $\sigma$ we compute the sliding window $dFC_w$ matrix from the simulated BOLD signal and quantify the “switching index” of the dFC as the variance of the upper triangle (**A**). For selected values of $(G,\sigma )$ we show the sliding window $dFC_w$ (**B**), edge-based $dFC_e$ (**C**), and the projection of $\textbf{r}(t)$ time series in the first two PCA components (**D**) annotated with corresponding fractional variance accounted for (VAF). In the working point around $G=0.54$ and intermediate values of $\sigma$ the system exhibits recurrence in the large-scale dynamics as captured by non-zero switching index, and reduction of dimensionality as captured in the increase in explained variance by the first PCA components and the asymmetry in the respective projection. For values of *G* below or above the working point, the system loses the fluidity property as reflected in the absence of the off-diagonal blocks on the dFC, and exhibits high-dimensional dynamics. Kolmogorov-Smirnov distance of the centered (mean-subtracted) distributions of the values of the upper triangle of the dFC computed from empirical and simulated resting state BOLD time series. The region of parameter space where the distributions are the closest overlaps with the region with high fluidity of dFC (**E**). The coincidence of the high fluidity of dFC and the similarity to empirical data is preserved across 100 subjects from the Human Connectome Project dataset (**F**).

In particular, we investigate the behavior of the system between two symmetrical limit cases in terms of the amount of afferent node coupling input: starting from the uncoupled system (invariant under node labeling), across the regime with broken symmetry (heterogeneous distribution of coupling input due to the asymmetrical connectome), up to a symmetrical saturated regime (high values of afferent input overriding local node dynamics, invariant under node labeling). To assess the impact of the symmetry breaking by the connectome, we simulated 10 minutes of spontaneous activity for a range of values of the coupling scaling parameter *G* and noise variance $\sigma$. The scaling parameter *G* multiplies in the networked model the afferent coupling at each of the nodes, while $\sigma$ is the variance of the driving noise at each of the nodes (see Section "Brain network model" for precise definitions). We then applied PCA to the source signal $\Psi (t)$ and dFC to the BOLD time series (Fig. 2), where the source signal $\Psi$ is the state vector of the system (see Section "Brain network model"). We used the variance accounted for (VAF) of the first two PCA components as an estimate for the dimensionality of the system’s dynamics in the state-space (Fig. 2D), and the *switching index* defined as the variance of the upper triangle of the dFC matrix as a measure of the fluidity of the system’s dynamics (Fig. 2A).

For low values of *G*, the system dynamics lacks recurrence (Fig. 2B,C), as shown by the low off-diagonal values of the dFC matrix, which captures the similarity of the FCs between distant time-points. Simultaneously, the dimensionality of the dynamics is high as reflected in the low variance explained by the first two PCA components (Fig. 2D). Note, that the explained variance for each of the two PCA components is equal to 1/*N* (in this case $N=84$ nodes of the network), and the projection in panel D reflects the independent rare switching of two nodes, each captured by one PCA component (see Fig. S4 for spatial maps of the PCA components).

Around the value of $G=G_{w}=0.540$ and $\sigma _w=0.030$ (working point) there is an increase of the dFC fluidity, as quantified by the switching index. In this regime, the system exhibits coordinated cascades of up- and down-state transitions. On the dFC matrix (Fig. 2B,C), the fluid regime is characterized by the on-diagonal nonzero blocks reflecting the time intervals of invariance of the FC, together with similarity across time (high off-diagonal nonzero blocks). At the same time, the variance explained by the first two components of the PCA increases substantially pointing to the decrease in dimensionality of the system dynamics (Fig. 2D).

Past the working point ($G>0.6$) the off-diagonal dFC correlation drops following the decline of the similarity of FC across time. This is accompanied by the decrease of the explained variance in PCA signifying an increase in the dimensionality of the spontaneous dynamics. No such decrease in dimensionality and increase in the fluidity of the dynamics is observed when the connectome is replaced by an all-to-all network (Fig. S1).

In addition, using Kolmogorov-Smirnov (KS) distance between the centered distributions of the values of the upper triangle of the $dFC_w$ in the empirical and simulated data, we have verified that the region of the parameter space where dFC is most similar to the one derived from empirical data overlaps with the region with the highest fluidity, (Fig. 2E). The centering was performed to mitigate the biases of the KS distance arising from differences in the mean $dFC_w$ values. Such difference can have many sources unrelated to our focus, such as fluctuations in the empirical data across sessions, preprocessing choices^51^, and the difference in the mean $dFC_w$ between empirical and simulated data due to the lack of e.g. physiological noise in the simulations. See Fig. S5 for the comparison of centered and non-centered cumulative distribution functions of $dFC_w$. Furthermore, this similarity is preserved across subjects, which we verified by comparing the maximum of the fluidity ($G |_{\max (\text {fluidity})}$) and the minimum of the KS distance ($G |_{\min (\text {KS})}$) in 100 connectomes of 100 subjects from the HCP dataset (Pearson $r=0.95$, $p<10^{-5}$, Fig. 2F). See Fig. S2 for the fluidity results for the full range of values of *G* for all subjects.

### Network dynamics

Before we delve into the characterization of the low-dimensional manifold, let us first describe the network dynamics in more detail. In particular, here we are looking for the mechanistic properties of the network neural mass model which gives rise to the observed fluid dynamics in the working point identified in the previous section.

**Fig. 3:**
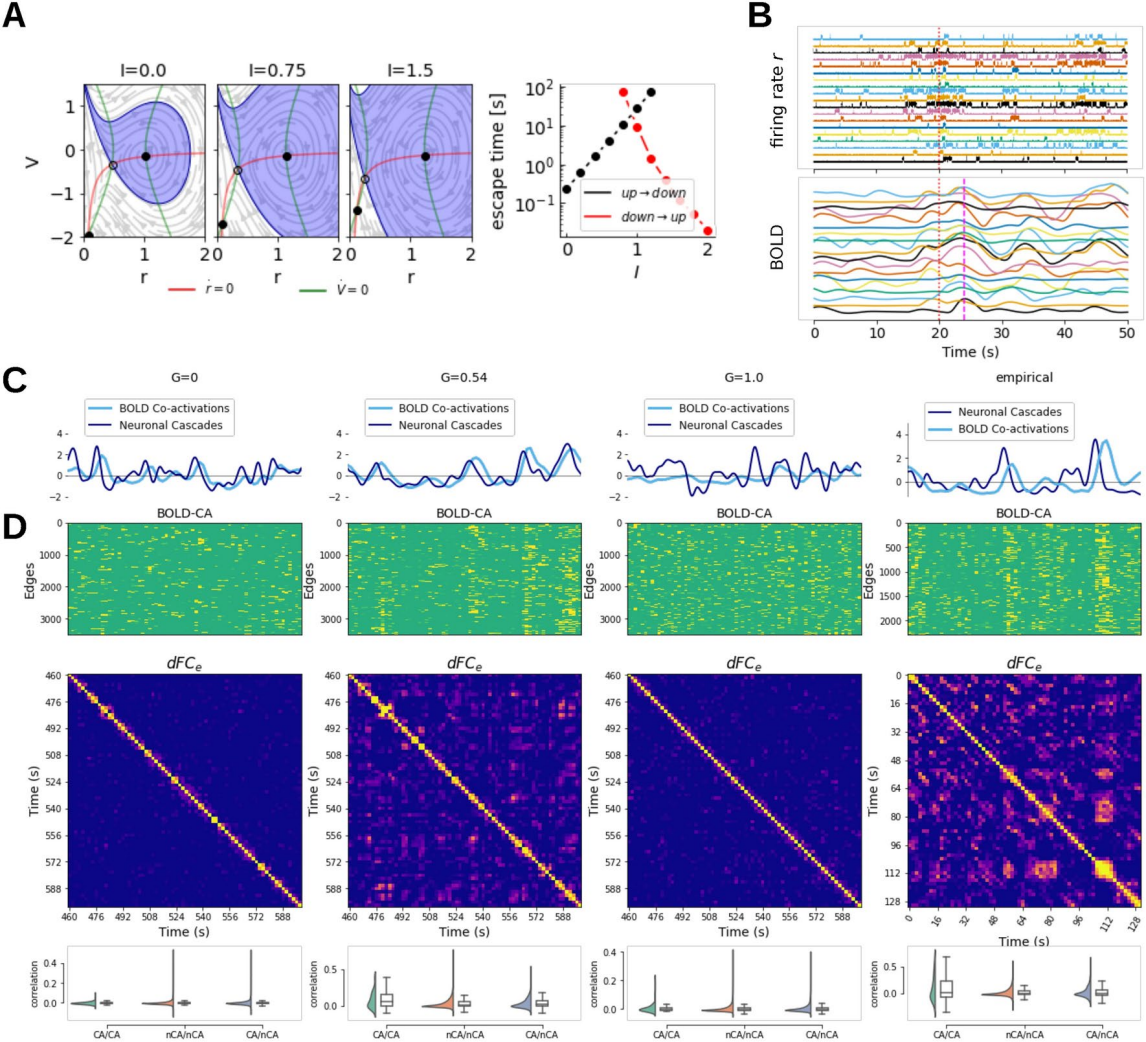
Network dynamics. (**A**) The network input *I* modulates the probability of a noise-driven transition between the down- and up-state by increasing the basin on the attraction of the up-state (blue area) in the phase-plane of firing rate *r* and membrane potential *v* with nullclines $\dot{r} = 0$ and $\dot{v} = 0$ shown in red and green respectively. (**B**) Example of a cascade—coordinated increase in activity translating to a delayed correlated peak in the BOLD signal. Below we compare the network dynamics in and outside the working point, and the empirical data. In both empirical data and the working point ($G=0.54$), the BOLD co-activations follow the neuronal cascades of firing rate *r* (simulated) and EEG (empirical) (**C**), and show distinct spatial profiles which are recurrent in time (**D**): edge time series on the (top panel) captures the spatial profiles of the co-activations, the similarity across time is captured by the $dFC_e$ matrix (middle panel), and the distributions of correlation between co-activation events (CA) and non-events (nCA) is compared (bottom panel).

For the MPR model, the dynamical profile of an isolated node in the bistable parametrization consists of an unstable fixed point (saddle-node) and two stable fixed points: down-state stable node and up-state focus (Fig. 3A). For an isolated node, varying the external input $I_i$ changes the size of the basins of attraction of the stable fixed points. This modulates the probability of switching between the two states when driven by noise as captured by the mean escape times (Fig. 3A, see Methods for more detail). For a connected node, the external input $I_e$ depends on the state of the neighboring nodes (see Equation 4), fluctuating as they transition between the up- and down-state. On the network level, given the right scaling of the network connections—the working point, this enables the sequences of up- and down-state switching at the fast timescale, and the co-fluctuation of the BOLD signal (Fig. 3B). When the coupling is too weak, the external input of all nodes is too weak to modulate the escape times enough to support the cascading effects, leaving the nodes effectively symmetric. Similarly, after the working point, the network input renders the basin of attraction of the downstate too small and the characteristic escape time too short to support the successive transitions to the down-state and the whole network becomes symmetric again, effectively locked in the up-state. The properties of the networked system are treated in detail in Section "Fixed point skeleton and structured flow".

To understand better the dynamical underpinning of the increase of fluidity of the dFC we assess the characteristics of the co-fluctuations of the BOLD signal and the cascades in the source signal. For the co-activations, we start from the edge time series which is defined as the pairwise dot product of z-scored BOLD signal (an average over the edge time series would correspond to the Pearson correlation). The correlation across time points yields the $dFC_e$ matrix capturing the recurrence of the edge configurations, and the root sum squared (RSS) over the edges at each time point captures the contribution of that particular time point to the overall functional connectivity (see Methods for more details). The time points crossing the 95th percentile threshold of the RSS are considered as strong co-activation events. The neuronal cascades^44^ are a measure of global level of deviation of the activity from the baseline, and are computed as a sum over regions of the binarized firing rate activity (at the threshold of 3 standard deviations). We compared these measures between the working point $G_w$, the disconnected system $G=0$, the strong network coupling regime $G>>G_w$, and the empirical data (Fig. 3C,D).

In the working point $G_w$ the co-activations include a large number of edges (Fig. 3D) and the RSS follows the number of cascades up to a short delay corresponding to the delay of the BOLD signal. Moreover, some of the strong co-activations re-occur partially in time as reflected in the non-zero elements of the $dFC_e$ matrix. The same profiles can be observed in the empirical data (Fig. 3C,D, right column), namely in the simultaneous EEG and fMRI recordings^52^. Additionally, the frequency of the co-activation events matches the empirical data in the working point $G_w$, but is significantly higher elsewhere (Supplementary Fig. S7). On the other hand, the characteristic spatial and temporal structure is lost outside the working point, that is either for the weakly coupled system ($G<<G_w$), or for too strong coupling ($G>>G_w$).

To quantify how the co-activation events contribute to the characteristic similarity across time, we compare the correlation of the edge vectors during the events, during the non-events, and between events and non-events (Fig. 3D, bottom row). As a result, we observe an increased similarity of the edge vectors during the events both in the empirical data and in the simulations in the working point $G_w$ as compared to the similarity of the edge vectors between events and non-events (t-test $p=0.01$ for simulated and $p<0.0001$ for empirical) or during non-events (t-test $p=0.01$ for simulated and $p<0.00001$ for empirical). Again, this property is lost for too weak ($G<<G_w$) or for too strong coupling ($G>>G_w$). Together, these results show, that the system has a similar dynamical profile in the working point $G_w$ as observed in the empirical data for the network-carried fluctuations on both the fast and slow timescales (as captured by *dFC* and cascades respectively).

### Manifold of the resting state and characteristic subspaces

Having characterized the dynamics of the system in the working point in terms of the cascades and co-fluctuations, we proceed with the description of the manifold on which this activity evolves. In particular, we aim here to identify the subspaces of the system state-space where the co-fluctuations occur, and then unfold the trajectories of the co-fluctuations in these subspaces.

**Fig. 4:**
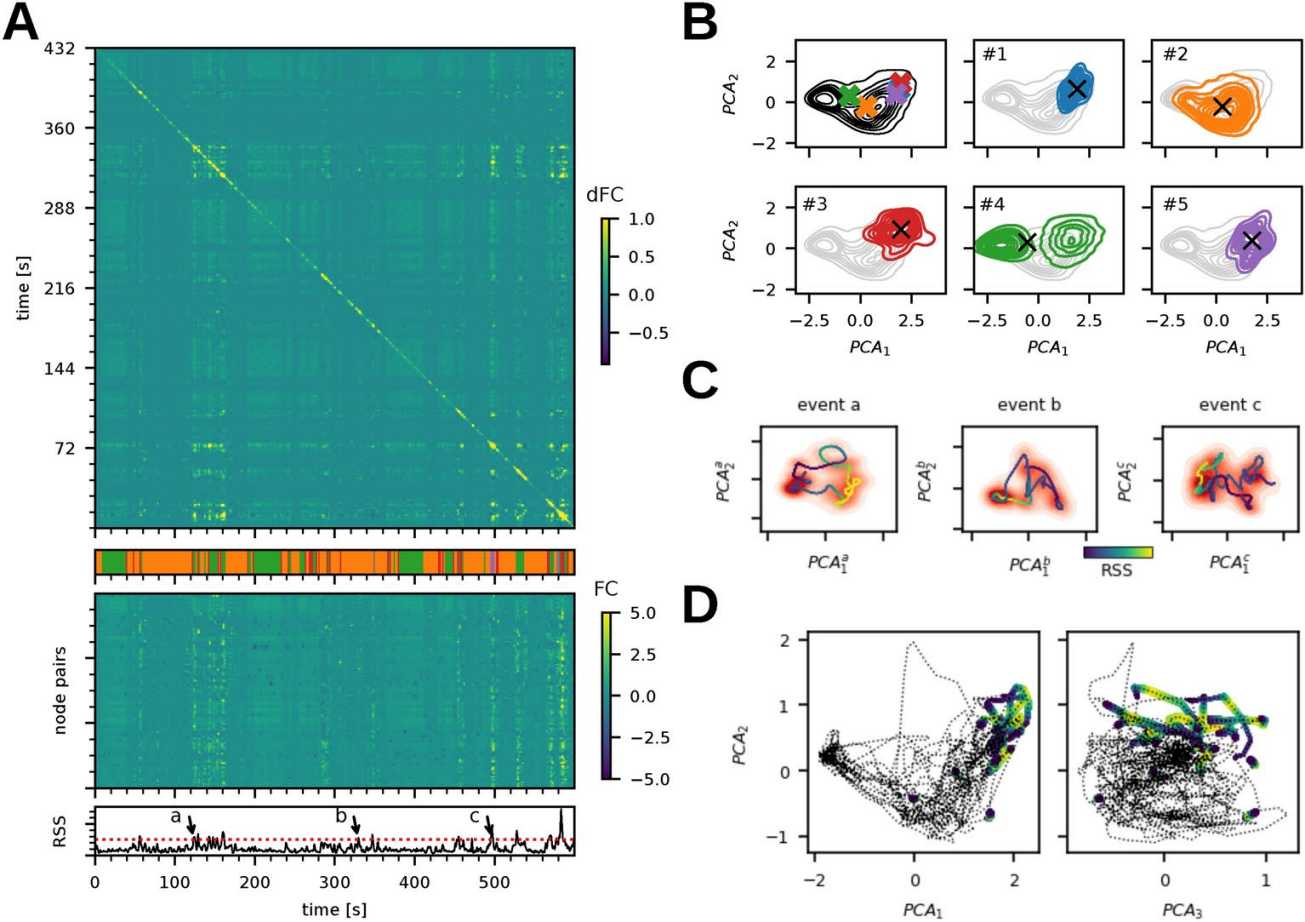
Manifold subspaces and characteristic dynamics. (**A**) The edge-based dynamic functional connectivity $dFC_e$ of a simulation of the model in the working point (top) shows the off-diagonal structure of similarity of the system’s activity across time. The edge time series (middle) shows the time evolution of the functional connectivity of the simulated BOLD signal between each of the node pairs and exhibits the characteristic co-activation events defined as time points with the root sum squared (RSS, bottom) crossing the threshold of 95th percentile. Dividing the edge-time series into 5 clusters (k-means, shown in the color bar under the dFC) has separated the event and non-event time points, and also differentiated the events based on their respective similarity. (**B**) The time intervals in $\textbf{r}(t)$ corresponding to the 5 clusters were selected; in the first panel, the centroids of the time points of the individual clusters are marked with a cross in the projection to the first two principal components of the whole time series, following panels show the projection of the $\textbf{r}(t)$ intervals of particular clusters. All time points are shown in aggregation as density plots. Cluster #2 captures the high-activity subspace, cluster #4 corresponds to the low-activity state, and the clusters #1, #3, and #5 capture the co-activation events. (**C**) Local trajectories in the manifold subspaces: the time series of the three example events (a, b, c, marked in panel A bottom) was projected to the first two components of PCA applied to each time segment individually. The red gradient denotes the dwell time of the system in the projected plane and shows a clear separation of the event subspace together with the rapid in- and out-transition. The smoothened trajectory marks the advance of the system through the event and out of it and is colored by the value of RSS (yellow at the peak of the event and blue at the beginning and the end). (**D**) The event trajectories on the manifold. The trajectory of the simulated BOLD signal is projected in the space defined by the first three PCA components with the events colored by the RSS value (yellow at the peak of the event).

To relate cascades and co-activations to the trajectories of the system in the 2N-dimensional state space, we first select time intervals with similar functional connectivity. Starting from the edge time series for the magnitude of co-fluctuations, we clustered the time points using k-means (k=5). This separated the high-activity intervals (majority of the nodes in the up-state), low-activity intervals (majority of the nodes in the down-state), and the co-fluctuation events (Fig. 4A).

Next, we identified the trajectories of the system underlying each cluster in the low-dimensional projection of state space. For each cluster, we have selected the corresponding time points in the state space of the system and projected them into the first two principal components of the PCA computed on the complete time series. We have observed that while the corresponding subspaces overlap partially in the projection (Fig. 4B, colors correspond to the clusters, all time points are aggregated to density plots), the activity within the clusters concentrates on different subspaces. This concentration in different subspaces is reflected in the distance between the centroids of the cluster time points in the PCA projection. We note that the PCA is here not used to define the manifold, but rather to provide a suitable space to analyze its properties. Similar results can be obtained with other embedding techniques such as the Isomap (^53^, see Fig. S6).

While the cluster activity overlaps in the projection in the components of the PCA computed from the whole time series, the co-activation trajectories become clearer by choosing different basis to span the low-dimensional space, that is, to compute the PCA from the time points corresponding to the individual co-activation events. To project the trajectories of the events observed at the slow timescale of the BOLD on the manifold, we have shifted the BOLD signal by the characteristic lag, and for each BOLD time point belonging to the cluster we selected the corresponding time points in $\textbf{r}(t)$, and convolved the resulting data with a Gaussian kernel to smoothen out the noisy fluctuations (see Methods for details). We then spanned the subspace corresponding to the first two PCA components of the co-fluctuation trajectory and overlaid the smoothed trajectory over the density plot of the full $\textbf{r}(t)$ time series. The density plots (shades of red, Fig. 4C) of the example events show a separation of the event subspace marked by the peak in the RSS (shown in yellow in Fig. 4C) on the smoothened trajectory from the rest of the manifold. The concentration of the dwell time away from the regions of the state space occupied before and after the event together with the rapid transitions in and out of the event suggest that the event subspace is relatively stable and that the intermediate states are less stable than the event subspace or the rest of the manifold and are visited only transiently. This stability also allows the system to dwell in event subspace long enough to cause significant peaks in the slow BOLD signal.

Although the linear embedding of the whole time series does not separate the event trajectories well when applied to the $\textbf{r}(t)$ time series, the event trajectories concentrate in the high-activity subspace spanned by the first two PCA components of the BOLD signal (Fig. 4D).

Together, these results chart the low-dimensional manifold of the system in the working point regime, associating the subspaces with specific flows. The fluid dynamics as characterized in the previous section then arise from the slow transitions between the low- and high-activity subspaces, where the latter supports the strong co-activation events which are reflected in the *dFC*.

### Fixed point skeleton and structured flow

To understand how the resting state manifold arises, we now go back to the equations defining the networked system to extract additional information besides what we have learned from analyzing individual realizations (trajectories) of the spontaneous activity in the previous sections.

We start by considering the uncoupled system, that is, the joint dynamics of the *N* populations (nodes) in the absence of any inter-population synaptic coupling. This uncoupled system’s phase flow is represented by $2^N$ stable fixed points that contain all possible combinations of the populations firing at either their low or high mean firing rates (down or up state, respectively). Starting from an initial condition (and in the absence of noise), the system will settle into the nearest accessible fixed point, a stable network state composed of a corresponding combination of regions in their up or down state. Thus, the dynamics of the uncoupled system in phase space can be thought of as being driven by a potential energy landscape with multiple stable local minima representing the stable attractor states of the network.

The dynamical effects of the symmetry breaking in the BNM are delineated by the topology of the connectome. The heterogeneity of the in-degree (total connectivity) of individual nodes of the network drives a variation in the relative positioning of the separatrices between the basins of attraction of the equilibrium points, mirrored in the variation of the corresponding projections onto the 2D phase planes of corresponding nodes (see Fig. 3A). In conjunction, connectivity strength and topology give rise to gradients in the relative attractiveness of the system’s equilibrium states. This attractiveness (or stability) can be quantified by the largest negative real eigenvalues obtained from the linearization of the system about the respective equilibrium state (linear stability analysis).

**Fig. 5:**
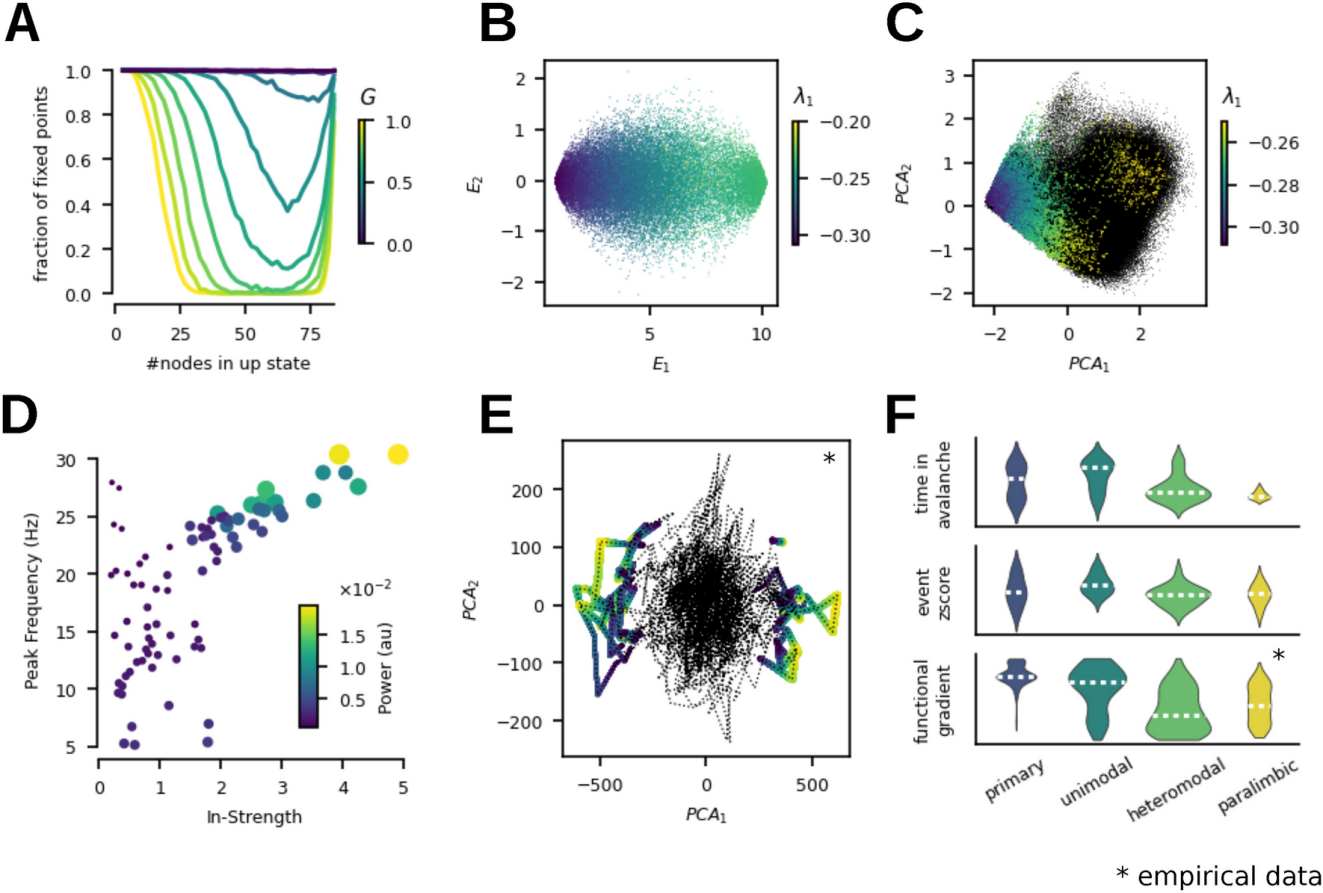
Mechanistic structure of the manifold and empirical observations. Panels ABC detail the fixed-point skeleton of the model, and panels DEF relate to the empirical observations. (**A**) Composition of the sampled stable fixed points in terms of a number of nodes in the up-state as a function of *G*, normalized to $G=0$. (**B**) Projection of the stable fixed points into the first two leading eigenmodes of the network Laplacian $E_1, E_2$, color-coded with the value of the largest eigenvalue in the linear stability analysis. (**C**) Fixed points (colored) derived by noise-free integration to equilibrium from the trace of a simulation (black) in the working point $G_w$, color-coded by the value of the largest eigenvalue $\lambda _1$. (**D**) Frequency peak in the simulated source activity of each region plotted against the node structural connectivity in-strength. (**E**) Empirical BOLD time series projected into the first two PCA components with the events colored by the RSS value (yellow in the peak). (**F**) Across the cortical hierarchy, the time spent in avalanches of the $\textbf{r}(t)$ time-series (top) decreases, as does the cumulative z-scored simulated BOLD from the event time-segments (middle). The spatial distribution of the principal functional gradient extracted from empirical fMRI is also aligned along the cortical hierarchy (bottom).

To map the complete manifold outside the simulated trajectories we sampled the stable fixed points for varying coupling scaling parameter *G* from the $2^N$ combinations of up- and down-states, and evaluated their stability (see Methods for more details). We found that the number of stable fixed points in the sample decreases with increasing *G*. This decrease is due to the loss of states with mixed composition of up- and down-state due to the bifurcation of the down state in nodes with high input (Fig. 5A). To map out the fixed points in a space defined by the properties of the structural connectivity (SC), we have employed the space spanned by the eigenvectors of the graph Laplacian which represent the structural connectome harmonics. In particular, the first eigenvector captures the shared (global) activity, whereas the second differentiates left-right asymmetry (Fig. S3). By projecting the $\textbf{r}$ component of the fixed points in the first two eigenvectors, we have confirmed that the thinning of the intermediate compositions is biased towards those with a higher number of nodes in the up-state (corresponds to the first Laplacian eigenvector $\lambda _1$). Additionally, the stability of the fixed points was inversely proportional to the number of nodes in the up-state, that is in the direction of $E_1$ the first eigenvector of the Laplacian (Fig. 5B).

To put this into the context of the simulated trajectories, we have next identified the fixed points around which the simulated trajectory evolved by taking initial conditions from the simulated trajectory and integrating the system without noise to the equilibrium. We have confirmed that in all instances the system reached a stable fixed point composed of a combination of up- and down-states and that the stability of these fixed points follows the same gradient in terms of the composition (Fig. 5C). We have also verified that the principal components of the simulated time series ($PCA_{sim}$) are indeed reflecting the switching between fixed points by applying the PCA to the sequence of the fixed points ($PCA_{fp}$) sampled from the simulated trajectory. Indeed, the correlation between the first two components of $PCA_{sim}$ and $PCA_{fp}$ was 0.97 and 0.95 for the first and second components respectively, and the variance accounted for of the $PCA_{fp}$ was only slightly higher at $VAF_{fp}=0.453$ ($VAF_{sim}=0.411$, see Fig. 2D).

The simulated trajectory didn’t explore the manifold completely, the number of visited stable fixed points (16,209), was about half of the 32,857 stable fixed points identified in the 50,000 randomly sampled initial conditions (Fig. 5A). So while the number of attractors is reduced compared to the disconnected G=0 case (48,160 fixed points from the 50,000 samples), the recurrent dynamics in the working point cannot be explained by the reduction of the number of attractors alone.

Furthermore, the nodes of the network exhibit a frequency gradient of the oscillations in the up-state (Fig. 5D). This gradient reflects the variability of the characteristic frequency in the up-state across nodes in the network. In the fixed-point state, if the nodes are treated as isolated systems with an input current term based on the existing network state, then

1 $$\begin{aligned} \begin{aligned} r_i^*&= r^* + \delta _i^r\\ v_i^*&= v^* + \delta _i^v \end{aligned} \end{aligned}$$

where $(r^*, v^*)$ are the symmetric fixed points of the network and $(\delta _i^r, \delta _i^v)$ are the excursions from the symmetric fixed-point and change according to the existing network state. These excursions depend directly on the in-strength of the *i*th node and the local states of its first neighbors.

Following linear stability analysis of the *i*th system around the fixed point (see Methods), the eigenvalues of the Jacobian matrix are given by

2 $$\begin{aligned} \begin{aligned} \lambda _{1,2}&= 2v_i^* \pm \sqrt{2Jr_i^* - 4\pi ^2 r_i^{*^2}}\\&= 2v_i^* \pm \sqrt{ 2Jr^* - 4\pi ^2 r^{*^2} - 2 \delta _i^r( 4\pi ^2r^* + 2\pi ^2\delta _i^r - J) } \end{aligned} \end{aligned}$$

From the above equation, we see that the frequency of oscillations in the up-state of the *i*th node increases proportional to $\delta _i^r$ given that all the involved terms under the square root are positive and that $J < 2 \pi ^2 r^*$ as already without the external input the up-state of the isolated nodes is a stable spiral with complex eigenvalues of the Jacobian. Therefore, the frequency of the up-state oscillations is proportional to the in-strength of the node, which we also observe in the simulation (Fig. 5D).

Furthermore, applying the PCA projection on the empirical BOLD time-series (Fig. 5E), we have identified a similar separation of the event trajectories in the global embedding as observed in the simulations (Fig. 4D). However, in the case of the empirical data the system exhibits both co-activations and co-deactivations as seen in the separation through the first PCA component. This discrepancy is one of the limitations of the model resulting from a lack of homeostatic or neuromodulatory mechanisms, as we discuss in more detail in the next section.

Lastly, symmetry breaking by the connectivity alone results in a spatial organization of the above-described flow which is aligned with empirically observed trends (Fig. 5F). For the spatial organization, we compare the features of interest against the cortical hierarchy^54^, which spans from unimodal (sensory, motor) to transmodal (higher-order) regions. This hierarchy is not only anchored in the anatomy and structural connectivity of the cortex but is also reflected in changes in the structure-function relationship^55^. In particular, the principal functional gradient, which is derived from the empirical fMRI functional connectivity matrices with the help of diffusion embedding, is also aligned along this axis^56^. For the main dynamical features of our system, per region, the time spent in avalanches (avalanche is a sustained deviation of the network activity from baseline level^57^, here defined as a set of nodes crossing a threshold of the z-scored $\textbf{r}(t)$) and the cumulative z-scored BOLD signal within events both decrease across the cortical hierarchy from the primary to paralimbic regions.

## Discussion

Using a combination of computational modeling and dynamical systems analysis we have provided a complete mechanistic description in terms of constituent entities and their causal activities leading to spontaneous co-activations and neuronal cascades in the brain’s resting state^44^. We showed how the breaking of the symmetry of the BNM’s connectivity in the interaction with the local node dynamics gives rise to the structured low-dimensional dynamics in the phase space and recurrent fluctuations of the functional connectivity (Fig. 2). These fluctuations arise from network-mediated cascades of up- and down-state switching and capture well the empirically found relationship between the strong co-activation events and the recurrence structure reflected by the functional connectivity dynamics (Fig. 3). The subspace accessible to the brain in this regime was charted and partitioned according to the characteristic flow associated with each partition (Fig. 4). Finally, this subspace and its associated flows arise from the rich fixed point structure of the system, and the differential stability of the nodes in these fixed points is not only reflected in the propensity to state switching that reflects the cortical hierarchy but also influences the dominant oscillation frequency (Fig. 5). In summary, these results support our hypothesis that the recurrent functional connectivity states of the resting state correspond to distinct subspaces on a low-dimensional manifold associated with distinct structured flows.

The central result of our work is that the symmetry breaking via the structural connectivity carves out an attractive subspace of all the possible states of the brain and that the flow on this manifold governs the characteristic dynamics of the brain (that is discarding the transient towards the manifold from arbitrary initial conditions). In this regime, the model captures the multistability and noise-driven exploration of the dynamic repertoire explored previously in computational studies^2,31,32,58,59^. The data features extracted from the time series provide a link between the empirical data and the model. Here, the functional structure in the brain is carried by the rare high amplitude co-fluctuation events as it was previously demonstrated in empirical fMRI data^19,21,60^, and in simultaneous EEG and fMRI measurements^44^. Similarly, a recent modeling study has shown the role of structural modules of the network in shaping the co-fluctuation events^61^, which is aligned with the brain network as the symmetry breaking gives rise to the low-dimensional dynamics.

The slow timescale fluctuations of the dynamic functional connectivity reflect the movement of the brain activity between the low- and high-activity subspaces of the manifold. The flow in the high-activity subspace supports the cascades, which in turn are reflected in the high-activity coactivations. This movement points to the multistable rather than metastable interpretation of the resting state dynamics. Namely, the metastable system is defined as free from attractors and stable fixed points^62^, and the emergent dynamics arise from transient visits of neighborhoods of unstable fixed points. As such, it does not support power-law fluctuations in the form of avalanches which are together with neuronal cascades core characteristics of the presented model^44^.

The movement between the low- and high-activity subspaces is also compatible with the observation of switching between low-amplitude incoherent and high-amplitude coherent states in empirical data^63^. Furthermore, the slow transitions between the high- and low-activity subspaces are compatible with the reports on the spontaneous infra-slow brain activity^64,65^ and the detailed reports on its spatiotemporal structure. For example, the slow traveling waves^22^ propagating along the principal gradient of cortical organization^56,66^ would provide a refined description of the trajectory through the manifold subspaces.

The attractive subspace of the low-dimensional manifold and the associated structured flow arise in the presented system from the changes in the fixed-point structure due to the irregular connectivity. In particular, the network input mediates the modulation of the escape times of the noise-induced transitions. These chain into domino-like sequences^67,68^, constituting the neuronal cascades. On a network level, our results elaborate the previous analytical results of increased entropy of the attractors in an Ising-spin network model for intermediate values of coupling strength^69^. The relationship to the dimensionality of the exhibited dynamics is such that for the low values of coupling strength *G*, where the Ising model is in the trivial state with all spins equal to 0, the model presented here is also driven by noise to the all-down state due to the significantly larger basin of attraction of the down-state, and the nodes make uncoordinated noise-driven excursions to the up-state reflected in the high-dimensionality of the dynamics. For high values of *G* the situation is the opposite, and for intermediate values of *G* the Ising model exhibits high entropy of attractors, which is in our case reflected in the available states organized in the low-dimensional manifold with the structured flow governed by the stability of these states.

Overall, the movement of the system through the subspaces of a low-dimensional manifold is in accordance with empirical and modeling results on recurrence and state clustering of resting-state fMRI BOLD recordings. Using clustering algorithms to partition the BOLD time series yields statistically similar and temporally recurrent whole-brain spatial coactivation patterns^18,45^ associated with specific dwell times and transition probabilities. However, compared to the clustering approaches applied to the BOLD time series, the SFMs allow us to refine the partitioning of the state-space in two aspects: we unfold the subspaces based on the similarity of the coactivations on the level of the BOLD signal, and we provide a detailed description of the flow of the system in these subspaces e.g. in terms of the cascades. The former is in line with the recent advances regarding the low-dimensional representation of meso-^70,71^ and macroscopic^72,73^ brain dynamics, but the latter describes the origin of those subspaces as constrained by the connectome. Interestingly, the clustering of phase-locking BOLD states^43^ leads to a very similar low-dimensional representation of the resting state dynamics to our approach, with a single dominant global phase-locked state and several transient partially phase-locked states related to functional networks. Similarly, by embedding the resting state data onto the task manifold extracted with the help of diffusion maps,^74^ found that resting-state time points concentrate in the task-fixation and transition subspaces, and only a minority of time points reach the cognitive subspaces of the task manifold. Here, we have not focused on the detailed analysis of the subspace transition probabilities and their correspondence to the empirical data, mainly because of the lack of other than noise-driven perturbations in the model, however, this remains a very intriguing point for future work, as the subspace transitions and their probabilities can be naturally expressed as a flow on the manifold.

The description of the structured flow addresses also the fast timescale by including the cascades, which we previously showed to relate to the co-activations observed in the BOLD signal^44^. In EEG literature, the spatiotemporal structure of the resting state dynamics is characterized with the help of microstates—sensor-level transient patterns lasting on average for 60-150 ms^75^. Attempts have been made to relate the microstates to BOLD activation clusters^76,77^, but identifying the sources generating the microstates with clustering or regression analysis has been challenging so far due to the unclear relationship between the broadband EEG activity and the BOLD signal fluctuations^78^. To advance we propose to reframe the question as a search for a shared manifold of the neuronal activity with specific slow and fast timescale characteristics which in turn are reflected in the EEG and the BOLD observables.

The manifold we describe is conceptually reminiscent of energy landscapes described in previous works^40,41^. However, previous energy landscape models, such as in^41^, implicitly assume energy minimization and thus, by construction, encode the hypothesis that the activation of two brain regions that are connected via a direct structural connection is more energetically favorable than that of two regions that are not directly connected. We make no such assumption here and, instead, the effective energy landscape emerges, in the form of a low dimensional manifold, out of the interplay of the non-linearity in the local neural mass model and the connectome, thus fully embracing the network impact, beyond the pair-wise interactions. In addition, previous energy landscape analysis^40^ assumed that the network changes only gradually by flipping one region at a time, and did not account for transitions in which several regions flip simultaneously. Treating the brain as a whole, the BNM that we presented here instead allows for such latter transitions of the system in state space, which may very well be due to strongly connected regions that can simultaneously influence their nearest neighbors during coactivation events.

It is worth pointing out that our framework covers only one part of the mechanisms that shape the brain’s manifold and the flow on it: the connectome. We have assumed identical parameters for each region, ignoring the known structural hierarchies^79^, which have been shown to improve the predictive value of the BNMs^63,80,81^. While we observed differential functional properties of the nodes across the cortical hierarchy^54^, we did not recover the exact spatial correspondence to the established functional gradients^56^. Neuromodulation and the subcortical drives^82^ are another missing aspect that similarly improves the performance of BNMs^83^, in this model for example by potentially extending the local dynamical repertoire with sustained both positive and negative activity fluctuations. However, both of these elements are not yet established in the framework of BNMs, as is the impact of the connectome^24^. Thus, our goal here is not to generate in silico observables that are as close as possible to the empirical one, which nevertheless differ a lot depending on the preprocessing, e.g. see^84^, but to focus on the generative mechanisms for the key data features across time-scales and neuroimaging modalities that render functional activity identifiable across subjects^12,13,85^.

Another major simplification in our model is related to the choice of the neuronal mass model. While the MPR model offers a direct link to spiking neurons and also can be tuned to give realistic gamma oscillations during high-firing activity, as we have done here, we operate the model in the bistable regime between high and low-firing rates. Even though such transitions can be observed during the awake state with the high-firing rate activity generally being accompanied by shorter periods of low firing rate (e.g.^86^), these dynamics are generally observed during different sleep stages^87^. Here the bistability is a necessary mechanism in the current configuration for observing the non-stationary dynamics as observed by dFC^32,44,88^.

A natural next step will be to extend the analysis to include the impact of the data-informed regional variance^89^ which is now reachable by TVB through EBRAINS^90^. Similarly intriguing direction for the extension of the framework presented here is in more refined inclusion of the subcortical structures, especially their impact through the neuromodulation. Notably, recent works^82,91,92^ exploring the role of the thalamus, locus coeruleus, and basal nucleus of Meynert in shaping the dynamical landscape of the cortical activity are already formulated in the dynamical systems’ language while incorporating carefully the detailed anatomical and cytoarchitectural knowledge. Integrating these advances in the SFM framework is a natural next step towards the original motivation of SFM, which is to link the mesoscopic neuronal activity to the behavior, as the intricate interactions between the cortex and the subcortical areas are one of the organizing principles of the underlying the biological mechanisms supporting behavior^93^.

Parcellation-induced variation of empirical and simulated brain connectomes at the group and subject levels is another issue that needs to be considered^94^. Nevertheless, we focus on general mechanisms without going on regional-level specificities, so the choice of parcellation should not play such a role.

And lastly, while we here describe the manifold and the associated flow in detail, we did not provide its compact formulation. The work presented here is the first necessary step towards obtaining such an object, which will lend itself not only to predictive validation with respect to the empirical data (across modalities and dynamical features), but can also serve as a basis for effective parametrization of inference models sidestepping many of the drawbacks of using the brain network models directly^95^.

In conclusion, our results show how low-dimensional dynamics arise from breaking the symmetry in the brain on the level of the connectome. Describing these dynamics as structured flows on manifolds allows us to bridge the gap between the observational measures and the state-space trajectories of the system. As such, this object is well suited for comparison across different models, scales, and neuroimaging modalities, and provides a means for integration of the diverse descriptions of the resting state dynamics. Moreover, we hypothesize that these are fundamental for the construction of task-specific flows and manifolds used in theories of brain function, such as predictive coding.

## Materials and methods

### Brain network model

Computational brain network model^96^ is used to simulate resting state activity under varying values of network coupling scaling parameter *G*. The dynamics of each of the network nodes were governed by the neural mass model (NMM) derived analytically as the limit of infinitely all-to-all coupled $\theta$-neurons^48^, namely for *i*-th node for the firing rate $r_i$ and membrane potential $v_i$ as:

3 $$\begin{aligned} \begin{aligned} \tau _c \dot{r}_i&= \frac{\Delta }{\pi \tau _c} + 2 r_i v_i ,\\ \tau _c \dot{v}_i&= v_i^2 + \eta - (\tau _c \pi r_i)^2 + J \tau _c r_i + I_i , \end{aligned} \end{aligned}$$

where $I_i$ is the input current, $\eta$ is the average neuronal excitability, *J* is the synaptic weight, $\Delta$ is the spread of heterogeneous noise distribution, and $\tau _c$ is the characteristic time.

The *N* nodes are then coupled with a connectome derived from empirical data as

4 $$\begin{aligned} I_i(t) = G \sum _j W_{ij} r_j(t-D_{ij}), \end{aligned}$$

where *G* is the network scaling parameter, $W_{ij}$ is the connection weight, $D_{ij}=L_{ij}/S$ is the delay caused by the propagation of the signal on a tract of length $L_{ij}$ with finite speed *S*. We picked the speed $S=2 m/s$ from the biologically plausible range^97^, and a connectivity matrix of a subject from the Human Connectome Project^49^ in the Desikan-Killiany parcellation^98^ with 84 regions including subcortical structures.

The equations 3 and 4 comprise the drift $a(\Psi ,t)$ in the stochastic delay differential equation formulation with linear additive noise reading:

5 $$\begin{aligned} d\Psi (t) = a(\Psi (t))dt + b(\Psi (t))dW(t), \end{aligned}$$

where $\Psi$ is the state vector $[\psi _1,...\psi _n]$ with $\psi _n=[r_n,V_n]$, *dW*(*t*) is a differential of a Wiener process with Gaussian increment with variance $\sigma ^2$, and $b(\Psi ,t)=1$ is the diffusion coefficient—here constant yielding the noise term additive.

The model was implemented in The Virtual Brain^47^ and equipped with BOLD forward solution comprising the Balloon-Windkessel model applied to the firing rate $\textbf{r}(t)$^50^.

The model parameters $\eta =-5.0$, $J=15.0$, $\tau _c=1.0$, and $\Delta =1.0$ were selected to set the nodes in the bi-stable regime in the absence of coupling^48^. We then varied the global coupling parameter *G* and the noise variance $\sigma$, and simulated 10 minutes of resting state BOLD activity with $TR=720ms$ after discarding 10 seconds of the initial transient from random initial conditions.

### Functional connectivity dynamics

To track the time-dependent changes in the functional connectivity, we compute the windowed dynamic functional connectivity $dFC_w$^32,99^ and edge dynamic functional connectivity $dFC_e$^44^. Starting from the regional BOLD time-series $B_n(t)$ for each node *n*, we compute functional connectivity matrices *FC*(*w*) for each time window $w=1...W$ defined as $B_n(t)|_{t_w }^{t_w + \tau }$ with window length $\tau =60s$ and window step size $t_{(w+1)}-t_w=2s$. Next we compute the $dFC_w$ matrix of order *W* as

6 $$\begin{aligned} dFC_w(i,j) = corr( FC(w_i)^\triangle , FC(w_j)^\triangle ), \end{aligned}$$

where $FC(w)^\triangle$ is the vectorized upper part of the *FC* matrix.

For the window-less $dFC_e$^44^ we start from the edge time-series^21^ defined as $E_{nm}(t)=z_n(t)z_m(t)$ for $n,m=1\dots N$ where $z_{n}(t)=\frac{B_n-\mu _n}{\sigma _n}$ is the z-scored BOLD time-series of a node *n*. The edge dynamic functional connectivity is then computed as a correlation between the edge vectors at each pair of time points $t_1$, $t_2$:

7 $$\begin{aligned} dFC_e(t_1,t_2) = corr( E_{nm}(t_1), E_{nm}(t_2) ). \end{aligned}$$

The co-fluctuation events (CF) are defined as time points in the edge time-series $E_{nm}(t)$ during which the root sum squared $RSS=\sqrt{\sum _{nm} E_{nm}^2(t)}$ crosses a given threshold, here chosen as 95th percentile. Time points where *RSS* is below the threshold are then labeled as non-events (nCF).

The avalanches were computed on the binary mask ${\textbf {a}}(t)$ on the ${\textbf {r}}(t)$ such that $a_i(t) = 1 \iff z(r_i(t)) > 3$ where $z(r_i(t)$ is the z-score of firing rate *r* of a node *i*. Neuronal cascades were computed as the sum of the binary mask *a*(*t*) over nodes, convolved with Gaussian kernel of the width of 1 BOLD TR, and downsampled to obtain the same resolution as the BOLD signal^44^.

The fluidity of the dynamic functional connectivity $dFC_w$ was evaluated with the *switching index*, defined as the variance of the values in the upper triangle of the $dFC_w$ matrix with the diagonal offset by the window size *W*.

8 $$\begin{aligned} s = Var(\{dFC_w(i,j) | i\le j-W\}) \end{aligned}$$

### Manifold subspaces

As a first step in the analysis of the local dynamics specific to a particular attractive subspace, we have extracted the time points belonging to these subspaces with k-means clustering applied to the edge time series $E_{nm}(t)$. We varied the number of clusters *k* and selected $k=5$ at which the co-fluctuation events were separated into distinct clusters.

To extract the segments of $\textbf{r}(t)$ corresponding to the $E_{nm}(t)$ time points we first estimated the BOLD signal lag $l={2500}\,\,{\hbox {ms}}$ as optimal peak-to-peak alignment with $\textbf{r}(t)$ smoothened by a Gaussian filter with same effective width ($\sigma =700$). Then for all BOLD time points in a given cluster *c* we selected the 2000 corresponding time points in $\textbf{r}(t)$ and concatenate these to get the fast timescale activity $\textbf{r}_c(t)$ in the subspace corresponding to cluster *c*. Each of the $\textbf{r}_c(t)$ was then projected to space spanned by the first two PCA components of the whole $\textbf{r}(t)$ time-series to evaluate how much of the overall state-space is covered by individual clusters.

The local trajectory for a given event *e* was computed by selecting interval $\textbf{r}_e(t)$ corresponding to BOLD time points above the RSS threshold and three time points before and after the event. Local $PCA^e$ of was then computed from $\textbf{r}_e(t)$, and the smoothened trajectory was computed by convolving $\textbf{r}_e(t)$ with a Gaussian filter ($\sigma =100$).

### Manifold sampling

To identify the fixed point scaffold of the manifold as traced by the trajectory resulting from integrating the Equation 5, we sample the segments from the simulated trajectory $(r_{i}(t),v_{i}(t))|_{t_s}^{t_s+\tau _{max}}$, and use them as initial conditions for integration of the deterministic interpretation of Equation 5, i.e. $d\Psi (t) = a(\Psi (t))dt$. From each such initial condition, we integrated the system to steady state equilibrium corresponding to a fixed point $(\textbf{r}^*, \textbf{v}^*)$.

The number of stable fixed points $(\textbf{r}^*, \textbf{v}^*)$ of the system with $G=0$ is $2^N$ reflecting all the combinations of up- and down-states of the *N* nodes. To sample the stable fixed points of the system with $G>0$ we solve repeatedly the system of equations:

9 $$\begin{aligned} \begin{aligned} 0&= \frac{\Delta }{\pi \tau _c} + 2 r_i^* v_i^* ,\\ 0&= v_i^{*2} + \eta - (\tau _c \pi r_i^*)^2 + J \tau _c r_i^* + I_i \end{aligned} \end{aligned}$$

using Newton-Raphson method with the initial conditions chosen randomly as a vector of up- and down-state fixed points of the isolated nodes, i.e. $(r_i^{*0},v_i^{*0})\in \{(r^*_\uparrow ,v^*_\uparrow ), (r^*_\downarrow ,v^*_\downarrow )\}, \forall i$ where $(r^*_\uparrow ,v^*_\uparrow )$ and $(r^*_\downarrow ,v^*_\downarrow )$ are the up- and down-state fixed points for the isolated node. For each initial condition $(\textbf{r}^{*0}, \textbf{v}^{*0})$ we then check if the corresponding solution of Equation 9 is equivalent up to the composition in terms of up- and down-states. If not, it is discarded, otherwise, we evaluate the stability of the found fixed point using linear stability analysis.

As a low-dimensional projection of the sampled manifold, we have used the two slowest eigenmodes of the structural connectivity. These are computed as eigendecomposition of the graph Laplacian $\textbf{L}= \textbf{W}- \textbf{I}$, that is $\textbf{L}\textbf{U}= \textbf{U}\mathbf \Lambda$, where eigenvalues $\lambda _k$ can be interpreted as structural frequencies and the eigenmodes $\textbf{u}_k$ as structural connectome harmonics^100^.

### Linear stability analysis

We perform a linear stability analysis to identify the fixed points obtained from the NR method. If each fixed-point $(\textbf{r}^*, \textbf{v}^*)$ is perturbed by $(\epsilon ^{\textbf{r}}, \epsilon ^{\textbf{v}})$ , then the evolution of the perturbations depend on the Jacobian matrix (*J*) and are given by:

10 $$\begin{aligned} \begin{bmatrix} \dot{\epsilon }^r_1 \\ \dot{\epsilon }^r_2 \\ \vdots \\ \dot{\epsilon }^r_N \\ \dot{\epsilon }^v_1 \\ \dot{\epsilon }^v_2 \\ \vdots \\ \dot{\epsilon }^v_N \end{bmatrix} = \begin{bmatrix} 2v_1^* & 0 & \dots & 0 & 2r_1^* & 0 & \dots & 0 \\ 0 & 2v_2^* & \dots & 0 & 0 & 2r_2^* & \dots & 0 \\ \vdots & \vdots & \ddots & \vdots & \vdots & \vdots & \ddots & \vdots \\ 0 & 0 & \dots & 2v_N^* & 0 & 0 & \dots & 2v_N^* \\ J - 2 \pi ^2 r_1^* & w_{12} & \dots & w_{1N} & 2v_1^* & 0 & \dots & 0 \\ w_{21} & J - 2 \pi ^2 r_2^* & \dots & w_{2N} & 0 & 2v_2^* & \dots & 0 \\ \vdots & \vdots & \ddots & \vdots & \vdots & \vdots & \ddots & \vdots \\ w_{N1} & w_{N2} & \dots & J - 2 \pi ^2 r_N^* & 0 & 0 & \dots & 2v_N^* \end{bmatrix} . \begin{bmatrix} \epsilon ^r_1 \\ \epsilon ^r_2 \\ \vdots \\ \epsilon ^r_N \\ \epsilon ^v_1 \\ \epsilon ^v_2 \\ \vdots \\ \epsilon ^v_N \end{bmatrix} \end{aligned}$$

The stability of a fixed point depends on the eigenvalues of the Jacobian evaluated at the fixed point. The fixed point is stable if all the eigenvalues of *J* are negative. Therefore, we numerically evaluate the largest eigenvalue of Jacobian for each fixed point and label the point as stable if its real part is negative.

### Fixed point sampling from simulated trajectory

From a given trajectory of the system given as 10 minutes of $\mathbf \psi (t)$ we have selected a restart point $t'$ each 50  ms (12000 starting points altogether). For each of the restart point $t'$ we extracted the segment $\Psi (t)|_{t'}^{t'-\tau _{max}}$ where $\tau _{max}$ is the length of the longest delay, and used as initial condition to an equivalent system to Equation 5 with $b=0$:

11 $$\begin{aligned} d\Psi (t) = a(\Psi (t))dt. \end{aligned}$$

Integrating this system to equilibrium yielded then for each restart point $t_r$ a fixed point $\Psi ^*=(\textbf{r}^*, \textbf{v}^*)$. The stability of each of the fixed points $\Psi ^*$ was then evaluated using the linear stability analysis as the largest eigenvalue of the respective Jacobian matrix.

### Escape time analysis

The switching behavior of a single node is driven by the stability of the up- and down-state fixed points in the presence of noise. We employed escape time analysis^101^ to measure the stability of these fixed points for a range of values of external input *I*. In detail, for a single node of the system given by Equation 3 we found the up- and down-state stable fixed points $(r^*,v^*)^\uparrow$ and $(r^*,v^*)^\downarrow$, and the unstable saddle-node $(r^*, v^*)^\times$. Next, we computed the separatrix between the two basins of attraction by integration of the model backward in time resulting in a closed curve $\omega$. To find the characteristic escape time for a fixed point $(r^*,v^*)$ we have integrated the system from the initial condition $(r_0,v_0)=(r^*,v^*)$ for a given value of *I* 100 times, measuring the time $t_E$ at which the trajectory crosses $\omega$ for the first time. The values of *I* were drawn from the range given by $[0,I_\text {max}]$ where $I_\text {max}=\max \{ I_i(t), \forall i\}$ is the largest value of $I_i$ encountered in the integration of the full system in the working point.

### Empirical data and spatial analysis

The principal functional gradient of the empirical fMRI data used in Section "Fixed point skeleton and structured flow" was computed from the group connectivity matrix of the Human Connectome Project dataset using the *BrainSpace* toolbox^102^. For a simulated resting state session with $G_w$, the time in the avalanche was computed for each node as the total time for which the $\textbf{r}_i(t)$ was above the threshold of 3 standard deviations, and the event z-score as a sum of z-scored BOLD signal in time points marked as events. The nodes were then grouped according to the cortical hierarchy^54^ projected to the Desikan-Killiany parcellation.

The previously published dataset of preprocessed simultaneous EEG and fMRI recordings of 15 subjects was used^52^. In summary, for 15 subjects (18–31 years) the following data used here were acquired: T1-weighted MRI and simultaneous EEG/fMRI resting state. The T1 image was used to parcellate the cortical gray matter according to the Desikan-Killiany atlas^98^, yielding 68 regions. This parcellation was then used to average the fMRI signals for each region and in subsequent EEG source reconstruction. The EEG data was first high-pass filtered at 1Hz, followed by MR imaging acquisition artifact correction using Analyser 2.0 (v2.0.2.5859, Brain Products, Gilching, Germany). The artifact-corrected data was then down-sampled to 200 Hz and low-pass filtered at 60Hz before correcting for physiological artifacts (ballistocardiogram, muscle activity). EEG source imaging was applied to the resulting cleaned sensor-level time series in order to estimate the activity on the cortical surface, which was then averaged according to the Desikan-Killiany atlas, resulting in 68 region-wise source time series. Details of the EEG and MRI processing steps are described in^52^. Additional quality control to reject residual movement artifacts was applied^44^ resulting in 30 artifact-free segments of EEG/fMRI time series (minimum duration 2 min) across the cohort.

A parcellation-based BOLD signals of a resting-state session from a subject from the Human Connectome Project^103^ were used to validate the separation of the events in the low-dimensional embedding. The data consisted of 1200 time points sampled at 720 ms in the Desikan-Killiany parcellation^98^ with 70 cortical regions.

## Supplementary Information

Supplementary Information.

## Data Availability

The simulated data generated during the current study are available from the corresponding author on reasonable request. The empirical dataset analyzed during the current study is available in the EBRAINS repository, https://doi.org/10.25493/F9DP-WCQ.
