# Supplementary material for: Symmetry breaking organizes the brain’s resting state manifold
Source: Sci Rep. 2024 Dec 30;14:31970. doi: 10.1038/s41598-024-83542-w (PMC11686292; doi:10.1038/s41598-024-83542-w)
Supplement: Supplementary file 1 — Supplementary Information. [file 41598_2024_83542_MOESM1_ESM.pdf]

Supplementary material

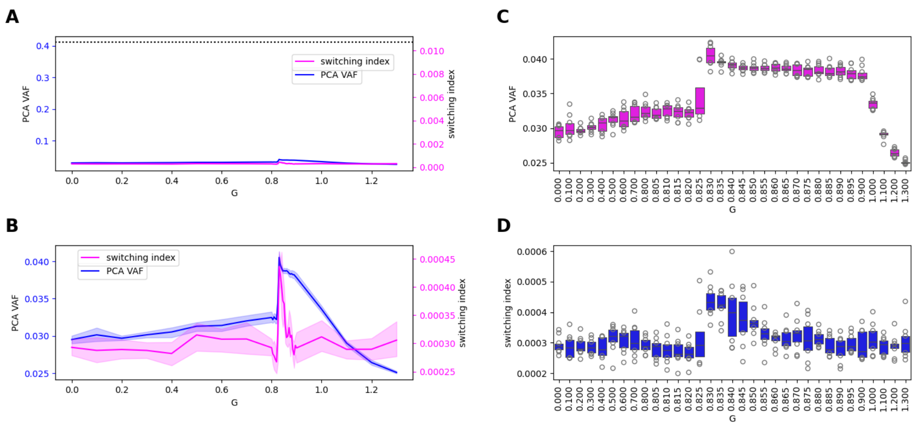

Figure S1: **Symmetric network coupling.** Replacing the connectome with a symmetric all-to-all network leads to the loss of both the low-dimensionality and fluidity of the spontaneous dynamics across the respective range of the network scaling parameter G. When compared to the maxima of the system coupled by the connectome (dotted line), the *Variance Accounted For* (VAF) of the first two PCA components of the $r(t)$ time-series and the fluidity (*switching index*) of the simulated BOLD signal stays at the level of the disconnected system (A). There is a small increase of both metrics around the value of $G = 0.830$, where the system switches discontinuously from the all-down to all-up symmetric dynamics (B, C, D), as there is no cascading activity after the initial transient. Error bars and confidence intervals capture variability for a given value of G over 10 noise realizations.

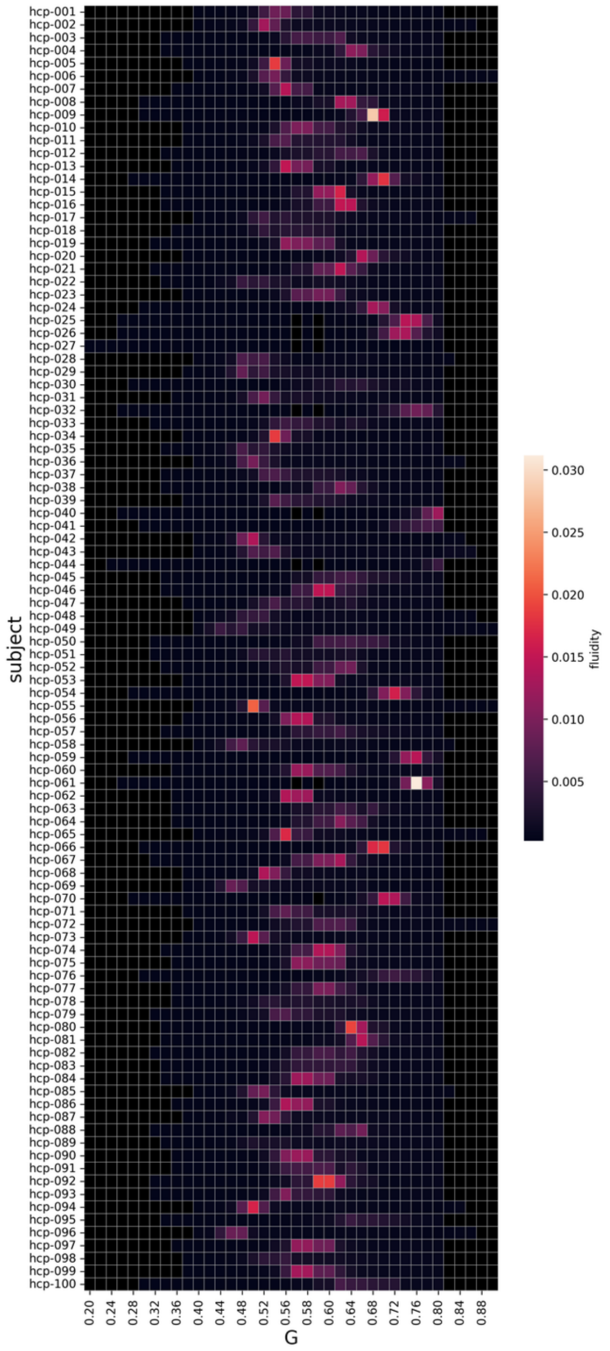

Figure S2: **Working point across subjects.** There is a single peak in the fluidity across subjects with respect to G.

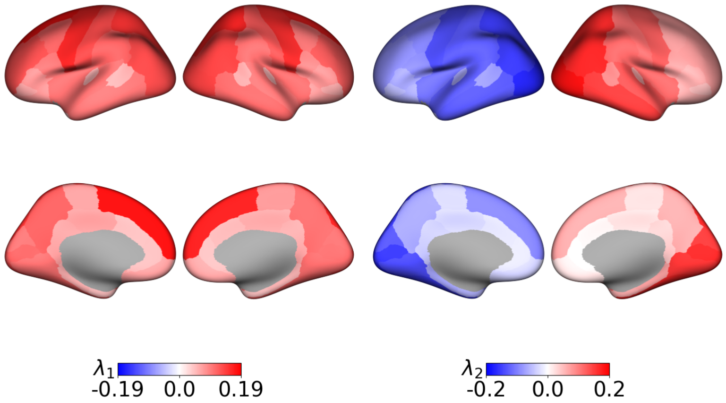

Figure S3: **Spatial maps of the Laplacian eigenmodes.** Maps for the first two eigenmodes λ_1 and λ_2 for the Laplacian of the structural connectivity of the subject from the HCP dataset in the Desikan-Killiany parcellation.

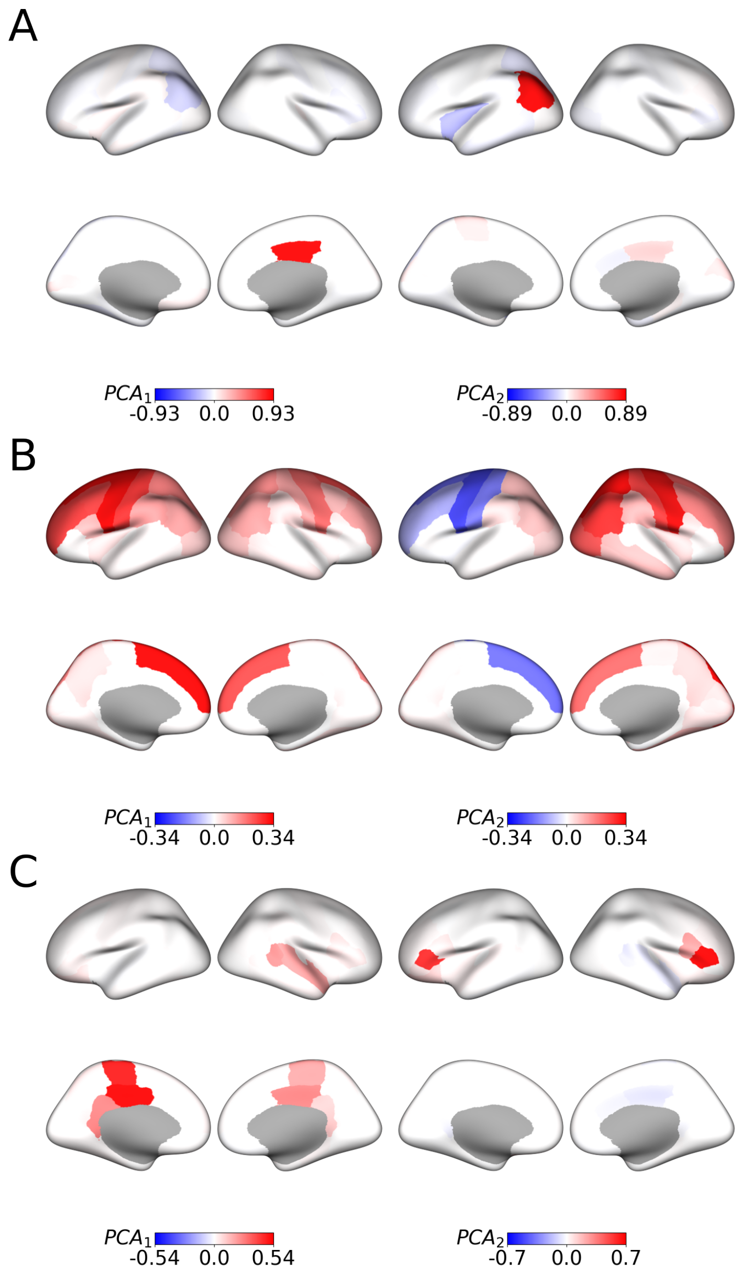

Figure S4: **Spatial maps of the Principal Components.** Maps for the weights of the first two PCA components of the $r(t)$ simulated time series for the disconnected system $G = 0$ (A), in the working point $G_w = 0.540$ (B), and beyond the working point $G = 0.8$ (C).

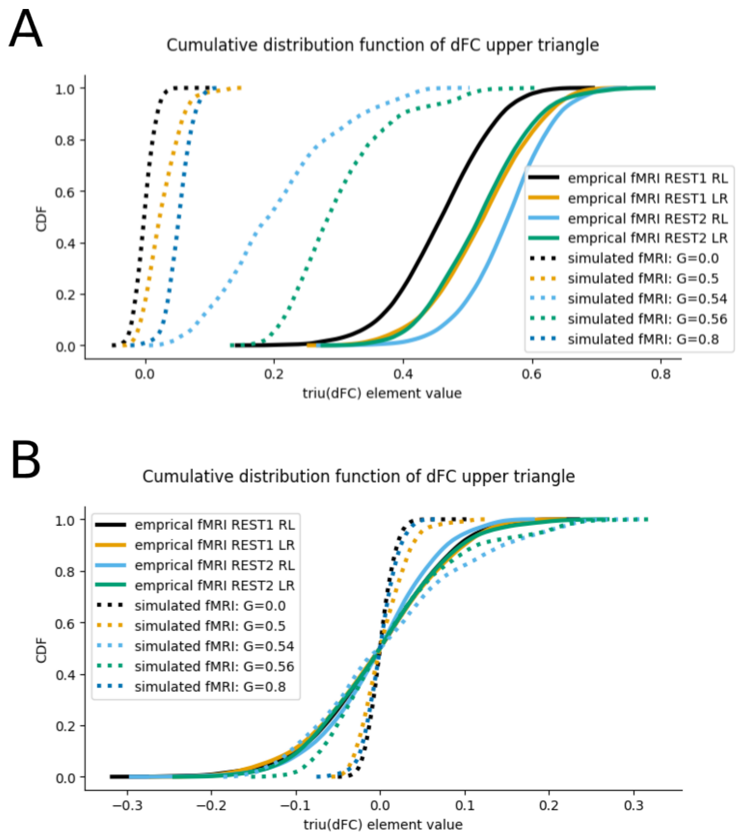

Figure S5: **Distribution of values in the dFC matrix.** Empirical cumulative distribution functions for the upper triangle of the dFC_w matrices of the simulated and empirical data of selected subjects from the HCP dataset without (A) and with (B) centering by subtraction of the mean. The *REST1* and *REST2* correspond to the two scanning sessions, *RL* and *LR* denote the two phase-encoding runs. See [104] for a detailed description of the protocol.

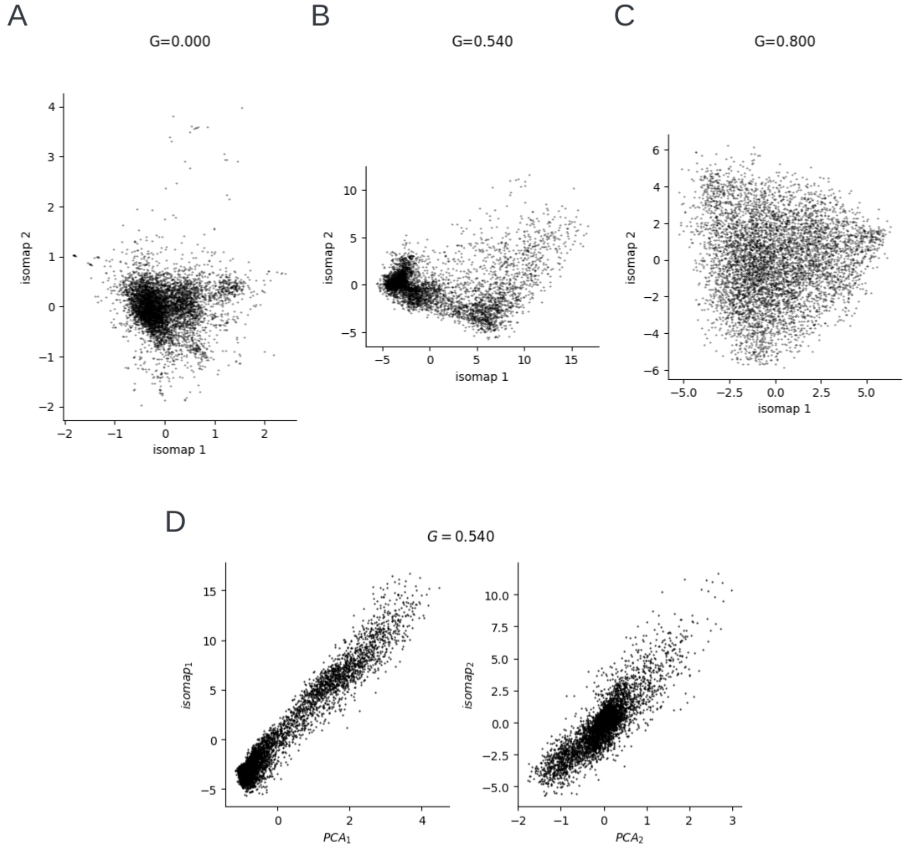

Figure S6: **Nonlinear embedding of the $r(t)$ time series.** The embedding of the $r(t)$ time-series using the *isomap* algorithm produces similar results as PCA used in the main text (see Figure 2D). Outside the working point (here $G = 0.0$ (A) and $G = 0.8$ (C)), the high-dimensionality of the dynamics prevents the extraction of meaningful embeddings. For the working point ($G = 0.540$ (B)), the nonlinear embedding separates the high- and low-activity subspaces same as the PCA, yielding a correlation of 0.98 and 0.86 for the first and second dimensions respectively (D).

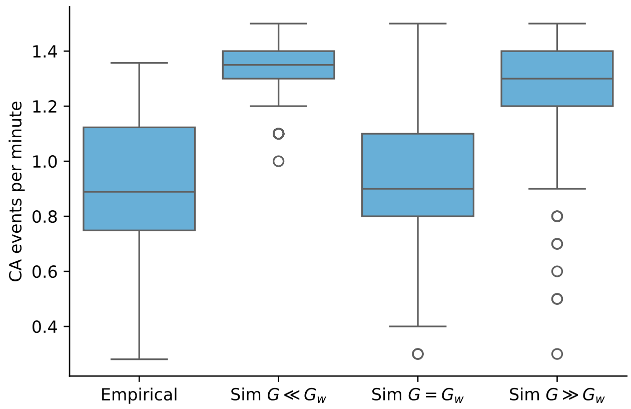

Figure S7: **Co-activation event frequencies.** Comparison of the number of co-activation (CA) events per minute in empirical and simulated BOLD time series. There is no statistically significant difference between the CA frequencies of the subjects of the empirical dataset [52], and the simulated 100 subjects in the individual working point $G = G_w$ ($p = 0.76$). Outside the working point (both $G \ll G_w$ and $G \gg G_w$), the frequency of CA events in the simulated data is significantly higher than both the empirical and the working point simulations ($p < 10^{-10}$).
